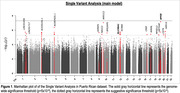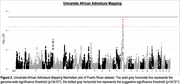# Genome‐wide association analysis and admixture mapping suggest an Alzheimer disease risk locus on chromosome 12 in a Puerto Rican cohort

**DOI:** 10.1002/alz.091600

**Published:** 2025-01-03

**Authors:** Bilcag Akgun, Briseida E. Feliciano‐Astacio, Joe Rivero, Kara L. Hamilton‐Nelson, Kyle Scott, Katrina Celis, Larry D. Adams, Jose Javier Sanchez, Glenies S Valladares, Concepcion Silva‐Vergara, Vanessa C Rodriguez, Pedro R. Mena, Patrice L. Whitehead, Michael B. Prough, Heriberto Acosta, Anthony J. Griswold, Clifton L. Dalgard, Katalina F McInerney, Jeffery M. Vance, Michael L. Cuccaro, Gary W Beecham, Farid Rajabli, Margaret A Pericak‐Vance

**Affiliations:** ^1^ John P. Hussman Institute for Human Genomics, University of Miami Miller School of Medicine, Miami, FL USA; ^2^ Universidad Central del Caribe, Bayamón, PR USA; ^3^ John P. Hussman Institute for Human Genomics, Miller School of Medicine, Miami, FL USA; ^4^ John P. Hussman Institute for Human Genomics, University of Miami Miller School of Medicine, Miami, FL, USA, Miami, FL USA; ^5^ Carribean Center for the study of memory and cognition, San Juan, PR USA; ^6^ Dr. John T. Macdonald Foundation Department of Human Genetics, University of Miami Miller School of Medicine, Miami, FL USA; ^7^ Department of Anatomy, Physiology, and Genetics, Uniformed Services University of the Health Sciences, Bethesda, MD USA; ^8^ University of Miami Miller School of Medicine, Miami, FL USA; ^9^ Department of Neurology, University of Miami Miller School of Medicine, Miami, FL USA; ^10^ John T. MacDonald Foundation Department of Human Genetics, University of Miami, Miami, FL USA; ^11^ 1501 NW 10th Avenue, Miami, FL USA

## Abstract

**Background:**

Hispanic/Latino populations are underrepresented in Alzheimer Disease (AD) genetic studies. The Puerto Rican (PR) population, a three‐way admixed (European, African, and Amerindian) population is the second‐largest Hispanic group in the continental US. We performed a genome‐wide association study (GWAS) in the PR population to identify novel AD susceptibility loci and characterize known AD genetic risk loci.

**Method:**

652 individuals (349 AD; 303 cognitively unimpaired), ascertained through the Puerto Rico Alzheimer Disease Initiative (PRADI), were included in the analyses. We performed GWAS on the Whole Genome Sequencing (WGS) dataset with a generalized linear‐mixed model adjusting for sex, age, and population substructure as fixed effects and genetic relationship matrix as a random effect. To infer local ancestry, we merged the target PR dataset with appropriate population samples from the HGDP and 1000G reference panels. Subsequently, we conducted univariate admixture mapping (AM) analysis. We also assessed the polygenic risk score (PRS) using the effect sizes from summary statistics from the non‐Hispanic White (NHW) study.

**Result:**

We identified a suggestive significant (p<5 × 10^‐6^) signal (rs11183403; P = 4 × 10^‐6^) within the *SLC38A1* gene on chromosome 12. Univariate African AM analysis identified one suggestive (p<4 × 10^‐5^) ancestral block located in the same region on chromosomes 12q13.1 (p = 7.2 × 10^‐6^). We replicated eight known AD loci– *APOE*, *ABCA7, CLU, FERMT2, GRN, PRDM7, SEC61G*, and *TREM2*. Admixture analysis revealed proportions of 68% European, 20% African, and 12% Amerindian in the PR cohort. NHW‐derived PRS has a good prediction power (AUC = 0.62) in the PR dataset.

**Conclusion:**

PR GWAS and AM identified a suggestive AD risk locus in the *SLC38A1* gene. This region overlaps with an area of linkage of AD in previous studies (12q13). The *SLC38A1* gene is associated with ischemic brain damage and its transcription is affected by amyloid‐beta peptide. Our study replicated 8 known AD loci previously identified in European studies and showed good predictive power with NHW‐derived PRS which is likely due to the high European background of the PR population. Including underrepresented populations in genetic studies is important for identifying health disparities and developing effective treatments for AD in all groups.